# Which medical interview skills are associated with patients’ verbal indications of undisclosed feelings of anxiety and depressive feelings?

**DOI:** 10.1186/s12930-016-0027-x

**Published:** 2016-02-28

**Authors:** Michiko Goto, Yousuke C. Takemura

**Affiliations:** Department of Education and Research in Family and Community Medicine, Mie University Graduate School of Medicine, 2-174 Edobashi, Tsu, Mie 514-8507 Japan; Department of Family Medicine, Mie University School of Medicine and Graduate School of Medicine, 2-174 Edobashi, Tsu, Mie 514-8507 Japan

**Keywords:** Family medicine, Medical interview, Depression, Anxiety, Communication

## Abstract

**Background:**

In medical practice, obtaining information regarding patients’ undisclosed “feelings of anxiety” or “depressive feelings” is important. The purpose of this study was to determine which interview skills are best suited for eliciting verbal indications of undisclosed feelings, for example anxiety or depressive feelings in patients.

**Methods:**

Our group videotaped 159 medical interviews at an outpatient department of the Department of Family Medicine, Mie University Hospital (Mie, Japan). Physicians’ medical interview skills were evaluated using a Medical Interview Evaluation System and Emotional Information Check Sheet for assessing indications of “feelings of anxiety” or “depressive feelings”. We analyzed the relationship between the interview skills and patients’ consequent emotional disclosure using generalized linear model (GLIM).

**Results:**

The usage of interview skills such as “open-ended questions” “asking the patient’s ideas about the meaning of illness” “reflection” and “legitimization” were positively associated with the number of anxiety disclosure, whereas “close-ended questions” and “focused question” were negatively associated. On the other hand, only “respect” was positively associated with the number of depressive disclosures, whereas “surveying question” was negatively associated.

**Conclusions:**

The results revealed that there are several interview skills that are effective in eliciting verbal indication of undisclosed “feelings of anxiety” or “depressive feelings”.

## Background

One of the important functions of the medical interview is to understand the mental status of patients. It is important that the physician checks the mental status of the patient during the medical interview, because patients often do not come to see physicians suspecting that they might have mental disorder [1, 2]. Paying attention to the patients’ emotions could not only contribute to early detection of psychiatric disease but is said to affect the patient’s satisfaction [3–5]. This approach will also shorten the consultation time and reduce the extra stress of the physicians [6]. However, in many cases, patients are said to only send indirect signals with no explicit emotional expressions [7] and show only an ambiguous response [8]. The cue expressed by the patient may be overlooked due to the physician’s low awareness [9, 10] or insufficient clinical skills due to the lack of training [11, 12]. Furthermore, even when they are encouraged by the physicians, many patients would feel embarrassed, hesitated or simply feel that they do not deserve to disclose emotions [13].

In recent years, many studies have been made on the relationship between the patients’ emotional response and the physicians’ interview skills to address these needs. Many of them have discussed that physicians’ communication skills should be employed to help patients’ express their emotions [6, 8, 14, 15]. However, most of the reports on the patients’ emotional response are made in the context of cancer treatment. Sufficient studies have not been made on the physicians’ skills and the patients’ emotional response during the common medical interviews like ones at primary care facilities.

The aim of the present study is to explore the association between physicians’ interview skills and patients’ disclosure about anxiety or depression in the primary care setting.

## Methods

### Participants and procedures

One hundred and fifty nine different new patients (74 males and 85 females) participated in this observational, cross-sectional study. They are patients at outpatient department of the Department of Family Medicine of large-scale hospital, Japan. The patients came from both rural and urban areas and were from a wide range of socioeconomic classes. All of the patients were over 15 years of age, and most patients generally had common medical problems such as hypertension, hypercholesterolemia, and diabetes. First, we checked whether this was a first visit and asked if the prospective participant were willing to participate, and if he or she declined, no further effort was made. Second, we gave them instructions about the purpose and meaning of the study and told them that they could decline from this study anytime when they want to. Third, we obtained each participant’s signature. Parental permission was obtained for patients whose age is less than 20 years old.

Each patient was interviewed by one of 26 family physicians, 98 family medicine residents, or 35 medical students having family medicine clinical clerkship. These interviews were videotaped for evaluation.

Informed consents were obtained from patients and physicians prior to any observation of the medical interviews. The research protocol of this study was approved by the Research Ethical Committee of the Mie University School of Medicine.

### Outcome measurements

The videotaped medical interview skills were reviewed by trained researchers using the Takemura Medical Interview Rating Scale (TMIRS) (Table 1) [16]. Some systems of the Roter interaction analysis system were used to build this scale [17]. “Feelings of anxiety” and “depressive feelings” were also operationally defined (Table 2). These items were all combined into a single sheet to allow us to count the skills that doctor’s used and patients’ emotional responses.

**Table 1:** Definition of each medical interview skill (Medical Interview Evaluation System)

Medical inter skill	Definition
Open-ended questions	An open-ended question invites the patient to use his or her own judgment in deciding what topics and problems to emphasize. These questions invite patients to describe their problems by using their own vocabulary and personal experience of their symptoms
Close-ended questions	A question which can be answered by Yes/No or a single word
Survey questions	A question after summarization (the summary) to survey the problem, and whether the patient has other problems or not
Focused questions	The question for understanding clearly the contents which a patient is going to tell and not a close-ended question but in-between open-ended questions and close-ended questions. In other words, questions which slightly limited the range of the answer. For example, “Where is the pain?”
Requests for feelings	Direct requests for the patient’s own feelings
Asking the patient’s ideas about the meaning of the illness	Directly asking the patient what he or she thinks could be causing the symptom
Asking the patient’s preferences about the examination	Directly asking the patient what kind of examination he or she would like to have or not have
Summarization	Attempts on the physician’s part to summarize the information settled to some extent that he or she has just received from the patient
Reflection	The physician’s statement of an observed feeling or emotion in the patient
Legitimization	An intervention that specifically communicates acceptance and validation of the patient’s emotional experience
Personal support	Letting the patient know that the doctor is there for the patient and wants to help
Partnership	Letting the patient feel a sense of partnership
Respect for patients	Respectful communication strategies, such as addressing the patient by name or giving affirmative comments

**Table 2:** Definition of feelings of anxiety and depressive feelings

Feelings of anxiety	The state of feeling nervous or worried that something bad is going to happen Linguistic expressions: scary, sleepless, anorexic, get scared, something wrong, apprehensive about, painful, concerned about, etc.
Depressive feelings	Unhappy, disappointed, or suicidal feelings Linguistic expressions: despair, feel like dying, boring, not fun, unhappy, not interesting in something, etc.

This scale was assessed for reliability. First, for each videotaped interview, an evaluator used the scale to evaluate the number of skills used by the physician. Second, the same evaluator did a second review a random selection of 25 interviews 2 months after the first review. The kappa value was calculated and found to be 0.93.

Interview length was considered as a confounder, because longer interview duration was presumed to positively influence our findings [18]. In addition, patients’ sex, sex matching between patient and physician, and affiliation (student, resident and family physician) were also obtained as other possible confounders.

### Statistical analyses

Demographic data were obtained first. Then, we used a generalized linear model (GLM) to assess the relationship between a physician’s use of these interview skills and verbal indications of feelings of anxiety or depression. Because our observation of physicians’ interview skills and patients’ emotional disclosures were recorded as count data, we modeled the relationship using the Poisson distributions and log link function. After the generalized linear model was estimated, we verified the variance inflation factors of each independent variable and the covariates (VIF). Finally, we evaluated goodness of fit of the estimated model using Akaike Information Criteria (AIC).

Statistical significance was set as *p* < 0.05 for all measures. We used R version 3.1.1 [19] for statistical analyses. Generalized linear model analyses were conducted using the “glm” function. There was no missing data.

## Results

Characteristics of patients and physicians are shown in Table 3. Additionally, the table shows basic statistics including mean value, standard deviations, and quartile values of each interview skill and emotional disclosure.

**Table 3:** Basic statistics of emotional disclosures, interview skills and covariates

Medical interview skill	Mean	SD		Quartiles				
				0 (%)	25 (%)	50 (%)	75 (%)	100 (%)
Demographic								
Sex of patients	Male: 74; female: 85							
Sex matching	Matched: 83; not matched 76							
Interview skills								
Open-ended questions	0.6	0.5		0	0	1	1	1
Close-ended questions	43.7	20.5		0	29	44	55	124
Focused questions	7.7	6.2		0	4	7	10	51
Survey questions	0.5	1.2		0	0	0	1	14
Request for feelings	0.1	0.4		0	0	0	0	3
Meaning of the illness	0.5	0.6		0	0	0	1	4
Preference	0.3	0.5		0	0	0	1	2
Summarization	0.4	0.5		0	0	0	1	2
Reflection	0.4	0.5		0	0	1	3	23
Legitimization	0.4	0.9		0	0	0	0	4
Personal support	0.1	0.5		0	0	0	0	4
Partnership	0.0	0.1		0	0	0	0	1
Respect for patients	2.2	2.1		0	1	2	3	13
Emotional disclosures								
Anxiety	3.4	4.6		0	1	2	5	41
Depression	0.3	0.9		0	0	0	0	32
Confounder								
Mean duration of interview (sec)	1356.5	730.1		133	834	1205	1753	4062

The estimated model for disclosures of anxiety is shown in Table 4, and the estimated model about that of depression is shown in Table 5. For the purposes of our analyses, only modified models will be considered.

**Table 4:** Estimated model of physician’s interview style on number of disclosure about anxiety by patients

Item	Crude^b^				Modified^a^			
	Estimate	SE	z value	*p*	Estimate	SE	z value	*p*
Intercept^c^	0.389	0.154	2.529	0.011	0.234	0.329	0.712	0.477
Open-ended questions	0.189	0.096	1.975	0.048	0.282	0.100	2.821	0.005
Close-ended questions	0.004	0.003	1.317	0.188	−0.008	0.004	−2.241	0.025
Focused questions	−0.008	0.008	−0.973	0.331	−0.018	0.009	−2.053	0.040
Survey questions	−0.044	0.031	−1.414	0.157	−0.041	0.032	−1.308	0.191
Requests for feelings	0.160	0.080	2.006	0.045	−0.004	0.080	−0.050	0.960
Meaning of the illness	0.207	0.069	2.995	0.003	0.157	0.070	2.256	0.024
Preference about examination	−0.077	0.093	−0.828	0.408	−0.033	0.092	−0.361	0.718
Summarization	−0.134	0.102	−1.317	0.188	−0.175	0.104	−1.683	0.092
Reflection	0.066	0.012	5.533	<0.001	0.066	0.013	5.195	<0.001
Legitimization	0.285	0.049	5.779	<0.001	0.205	0.051	4.014	<0.001
Personal support	0.009	0.092	0.093	0.926	−0.041	0.094	−0.435	0.663
Partnership	−0.982	0.593	−1.654	0.098	−0.543	0.607	−0.895	0.371
Respect for patients	0.070	0.024	2.899	0.004	−0.002	0.028	−0.079	0.937

Model was estimated using generalized linear model. Dependent variable was number of disclosure about anxiety

*SE* standard error

^a^Patient’s sex, physician’s title, sex matching between the patient and the physician, and duration of interview were modified as covariates. AIC was 768.85

^b^Akaike information criteria (AIC) was 810.00. AIC is an index of goodness of fit for the estimated model by generalized linear model

^c^Intercept is the value of dependent variable when all the independent variables were zero

**Table 5:** Estimated model of physician’s interview style on number of disclosure about depression by patients

Item	Crude^b^				Modified^a^			
	Estimate	SE	z value	*p*	Estimate	SE	z value	*p*
Intercept^c^	−2.478	0.440	−5.630	<0.001	−4.596	0.992	−4.634	<0.001
Open-ended questions	−0.458	0.231	−1.984	0.047	−0.379	0.258	−1.469	0.142
Close-ended questions	0.014	0.008	1.793	0.073	−0.008	0.009	−0.924	0.355
Focused questions	0.029	0.012	2.379	0.017	0.004	0.013	0.325	0.745
Survey questions	−0.089	0.049	−1.825	0.068	−0.115	0.052	−2.192	0.028
Requests for feelings	0.622	0.156	3.978	<0.001	0.049	0.158	0.313	0.754
Meaning of the illness	0.129	0.174	0.742	0.458	−0.065	0.194	−0.334	0.738
Preference about examination	−0.559	0.242	−2.308	0.021	−0.085	0.230	−0.367	0.713
Summarization	−0.008	0.264	−0.031	0.975	−0.179	0.297	−0.605	0.545
Reflection	−0.010	0.030	−0.338	0.736	0.009	0.035	0.267	0.789
Legitimization	0.350	0.142	2.462	0.014	0.007	0.173	0.039	0.969
Personal support	0.068	0.150	0.454	0.650	−0.218	0.164	−1.331	0.183
Partnership	−15.362	828.283	−0.019	0.985	−15.020	982.600	−0.015	0.988
Respect for patients	0.344	0.053	6.523	<0.001	0.215	0.065	3.293	0.001

Model was estimated using generalized linear model. Dependent variable was number of disclosure about depression

*SE* standard error

^a^Patient’s sex, physician’s title, sex matching between the patient and the physician, and duration of interview were modified as covariates. AIC was 286.96

^b^Akaike information criteria (AIC) was 322.39. AIC is an index of goodness of fit for the estimated model by generalized linear model

^c^Intercept is the value of dependent variable when all the independent variables were zero

Patients’ disclosures of anxiety were positively associated with “open-ended questions” “legitimization” “reflection” and “asking the patient’s idea about the meaning of the illness (meaning of the illness)”. Conversely, they were negatively associated with “close-ended questions” and “focused questions”. As none of the interview-skills and covariates showed VIF above 10 (data not shown), multicollinearity which was found to be within acceptable norms [20–23].

Patients’ disclosures of depression were positively associated with “respect for patients” and negatively associated with “survey questions”. As with the result for disclosures of anxiety, multicollinearity was found to be within acceptable limits, with no VIF above than 10 (data not shown).

We compared the respective association strengths of the various independent variables. “Open-ended questions” and “legitimization” were found to have the strongest association with respect to indications of undisclosed “feelings of anxiety”. “Close-ended questions” and “focused question” were also significant; however, their respective association strengths were close to zero. As for depression, “survey questions” and “respect for patients” were the only notable associations.

## Discussion

There were differences in which skills were effective for eliciting feelings of anxiety and depression clearly.

“Open-ended questions” “meaning of the illness” “reflection” and “legitimization” had a positive association with indications of “feelings of anxiety”.

“Open-ended questions” were effective for eliciting patients’ feelings of anxiety, but “close-ended questions” or “focused questions” were not. A lot of previous studies recommended using the open-ended questions to soliciting patients’ information [24–26] and the result of this study is in agreement with these investigations.

Previous study showed that “reflection” and “legitimization” were associated with patient satisfaction [16]. Another study also identified a significant positive association between the amount of information elicited from patients and patient satisfaction [27]. Furthermore, an investigation also indicated that patient satisfaction is associated with discussion of emotional distress [3, 4, 28]. This study might provide an explanation as to why “reflection” and “legitimization” have been shown to increase patient satisfaction, as enhanced disclosure has been shown to increase patients’ satisfaction.

“Meaning of the illness” was effective for eliciting patient’s feelings of anxiety, but “close-ended questions” and “focused questions” might conceal it. That results are in accordance with the previous investigation that patients are more likely talk about psychosocial issues in the atmosphere created when a physician uses less close-ended questions and less dominating [3, 4]. These results may show it is difficult for patients to express their anxieties when the patients were asked to verbalize the feeling directly by “close-ended questions” or “focused questions”.

In this study, “respect for patients” had positive association with indications of feelings of depression. Previous research showing that patients were more willing to divulge personal information; such as therapeutic, lifestyle, and psychosocial information to physicians who show respect supports the result of this study [29]. Cape and McCulloch also explored patients’ reasons for not presenting emotional problems in medical interviews and they found that 45 percent of reasons related to psychological embarrassment or hesitation to trouble the general practitioner or family physician [13]. Their result shares similarity in our result.

## Strength and limitations

Strength of this study is the use of an objective method to assess medical interview skills and patients’ emotion. Conversely, there are several limitations. First, there may be other confounding factors not measured in this study; for example, other relevant patient background information, non-verbal communications, and so on. Second, it is difficult to evaluate subjective information such as mental feelings objectively.

## Conclusions

This study reveals that several interview skills including “open-ended questions” “legitimization” would be effective in eliciting undisclosed “feelings of anxiety”. On the other hand, “respect for patients” was associated with undisclosed “depressive feelings”.

## References

[1] Robinson JW, Roter DL (1999). Psychosocial problem disclosure by primary care patients. Soc Sci Med.

[2] Ansseau M, Dierick M, Buntinkx F, Cnockaert P, De Smedt J, Van Den Haute M, Vander MD (2004). High prevalence of mental disorders in primary care. J Affect Disord.

[3] Bertakis KD, Roter D, Putnam SM (1991). The relationship of physician medical interview style to patient satisfaction. J Fam Pract.

[4] Levinson W, Roter D (1995). Physicians’ psychosocial beliefs correlate with their patient communication skills. Gen Intern Med.

[5] Blanch-Hartigan D (2013). Patient satisfaction with physician errors in detecting and identifying patient emotion cues. Patient Educ Couns.

[6] Mjaaland TA, Finset A, Jensen BF, Gulbrandsen P (2011). Physicians’ responses to patients’ expressions of negative emotions in hospital consultations: a video-based observational study. Patient Educ Couns.

[7] Suchman AL, Markakis K, Beckman HB, Frankel R (1997). A model of empathic communication in the medical interview. JAMA.

[8] Del Piccolo L, Saltini A, Zimmermann C, Dunn G (2000). Differences in verbal behaviors of patients with and without emotional distress during primary care consultations. Psychol Med.

[9] Tiemens BG, Ormel J, Simon GE (1996). Occurrence, recognition, and outcome of psychological disorders in primary care. Am J Psychiatry.

[10] Kessler D, Lloyd K, Lewis G, Gray DP (1999). Cross sectional study of symptom attribution and recognition of depression and anxiety in primary care. BMJ.

[11] Gask L, McGrath G, Goldberg D, Millar T (1987). Improving the psychiatric skills of established general practitioners: evaluation of group teaching. Med Educ.

[12] Gask L, Goldberg D, Lesser AL, Millar T (1988). Improving the psychiatric skills of the general practice trainee: an evaluation of a group training course. Med Educ.

[13] Cape J, McCulloch Y (1999). Patients’ reasons for not presenting emotional problems in general practice consultations. Br J Gen Pract.

[14] Hsu I, Saha S, Korthuis PT, Sharp V, Cohn J, Moore RD, Beach MC (2012). Providing support to patients in emotional encounters: a new perspective on missed empathic opportunities. Patient Educ Couns.

[15] Adams K, Cimino JE, Arnold RM, Anderson WG (2012). Why should I talk about emotion? Communication patterns associated with physician discussion of patient expressions of negative emotion in hospital admission encounters. Patient Educ Couns.

[16] Takemura YC, Atsumi R, Tsuda T (2008). Which medical interview behaviors are associated with patient satisfaction?. Fam Med.

[17] Roter DL (1991). The Roter method of interaction process analysis.

[18] Badger LW, deGruy FV, Hartman J, Plant MA, Leeper J, Ficken R, Maxwell A, Rand E, Anderson R, Templeton B (1994). Psychosocial interest, medical interviews, and the recognition of depression. Arch Fam Med.

[19] R core team. R: a language and environment for statistical computing. R foundation for statistical computing. Vienna Austria. http://www.R-project.org. Accessed 25 Aug 2014.

[20] Marquardt DW (1970). Generalized inverses, ridge regression, biased linear estimation, and nonlinear estimation. Technometrics.

[21] Neter J, Wasserman W, Kutner MH (1989). Applied linear regression models.

[22] Kennedy P (1992). A guide to econometrics.

[23] Hair JF, Anderson RE, Tatham RL, Black WC (1995). Multivariate Data Analysis.

[24] Roter DL, Hall JA (1987). Physicians’ interview styles and medical information obtained from patients. J Gen Intern Med.

[25] Maguire P, Faulkner A, Booth K, Elliott C, Hillier V (1996). Helping cancer patient’s disclose their concern. Eur J Cancer.

[26] Cole SA, Bird J (2000). The medical interview: the three-function approach.

[27] Takemura YC, Liu J, Atsumi R, Tsuda T (2006). Development of questionnaire to evaluate patient satisfaction with medical encounters. Tohoku J Exp Med.

[28] Gross R, Brammli-Greenberg S, Tabenkin H, Benbassat J (2007). Primary care physicians’ discussion of emotional distress and patient satisfaction. Int J Psychiatry Med.

[29] Cape J, Geyer C, Barker C, Pistrang N, Buszewicz M, Dowrick C, Salmon P (2010). Facilitating understanding of mental health problems in GP consultations: a qualitative study using taped-assisted recall. Br J Gen Pract.

